# Selection shapes diverse animal minds

**DOI:** 10.1098/rstb.2024.0108

**Published:** 2025-06-26

**Authors:** Ellouise Leadbeater, Alex Thornton

**Affiliations:** ^1^ Departement of Genetics, Evolution and Environment, University College London, London, UK; ^2^ Centre for Ecology and Conservation, University of Exeter, Penryn, Cornwall, UK

**Keywords:** animal cognition, learning, memory, comparative cognition

## Abstract

This special issue explores the evolution of cognitive diversity across the animal kingdom, challenging the value of approaches that have a basis in human psychology in favour of a perspective that embraces the multiple evolutionary endpoints that natural selection has produced. This approach centres around the key role of sensory processing as integral to cognition, rather than as something to be disentangled from it; around the relevance of so-called ‘simple’ processes; around the constraints that shape evolutionary trajectories and around the nonlinear nature of the selective environment. The field is advancing through approaches that span neuroscience, theoretical modelling, field studies, population genetics and more. By reframing cognition as a set of solutions to the natural world’s many challenges, we move closer to understanding animal minds as reflections of the numerous ways life has evolved to make use of information.

This article is part of the Theo Murphy meeting issue ‘Selection shapes diverse animal minds.’

## 1. Introduction

The concept of a linear scale of evolution with primates at the top is rejected by modern evolutionary biology, which recognizes all extant organisms as sitting at the pinnacle of their own evolutionary axis, and has done so since the time of Darwin. And yet, even Darwin was less firm in this assertion when considering the evolution of animal minds, which he discussed in terms of similarity to humans rather than a diversity of pathways to different endpoints: ‘The difference in mind between man and the higher animals, great as it is, certainly is one of degree and not of kind’ [1]. Perhaps it is because comparative cognition has its roots in psychology—a field that has the human mind as its core focus—that remnants of this perspective have lingered in considerations of the evolution of cognitive diversity [2]. A focus on those traits that define human behaviour makes sense when the aim is to understand the evolutionary trajectory that the human brain and the human mind have followed. Yet that trajectory is but one of many millions, and in this special issue, we are interested in the many *different* trajectories along which cognitive^1^ evolution might proceed across the animal kingdom. We are interested in how those trajectories are built, the selective pressures that drive them, the forces that shape and constrain them and the diverse endpoints that result. We find those species that do not excel at primate-typical cognitive tasks to be as informative as those that do. We are not comfortable with a view of supposedly ‘simple’ processes such as associative learning, sensitization and habituation as a basic departure point on an evolutionary ladder, because the networks that are built from such processes are part of the source material for much of the cognitive variation that exists within the animal kingdom [4–6]. In short, we are interested in the diversity of animal minds.

### (a) What are the traits that selection acts upon?

The issue begins with a thought-provoking perspective by Barton & Barrett [7], who question one of the fundamental tenets of comparative psychology: the idea of internal representations that are the basis for cognitive processes such as theory of mind, episodic memory or metacognition. They argue that a view of representational processes as entities that could be isolated from sensory–motor processing derives from a time before the realities of neural connectivity were available. Instead, they suggest that a framework that could genuinely capture cognitive evolution would be one that incorporated sensory–motor adaptations rather than attempting to control for them. In other words, animals with very different sensory systems and morphology may show convergent behaviours (e.g. behaviours that indicate a memory of what took place, where and when), but this should not be shoehorned into a human-focused framework as evidence of a shared trait (e.g. episodic memory) because those species-specific sensory–motor adaptations are fundamental parts of that trait that should be encompassed by research agendas. The argument that animal minds are not well characterized by whether they possess a list of concepts inherited from a historical literature grounded in constructs from human psychology is a refreshing challenge to established wisdom.

The following contribution by Lotem & Halpern [8] offers a valuable way to visualize how sensory–motor adaptations can become central to cognitive processes. In their theoretical framework, stimuli or events experienced simultaneously by an animal—such as the scent and colour of a flower for a bee, or two phonemes forming a word for a human—are treated as individual nodes in a network. These nodes can become linked if they are activated at the same time, but only under specific conditions (e.g. repeated co-occurrence) that are governed by learning parameters shaped by natural selection. In this model, two key components work together: sensory and attentional filters that determine whether nodes are activated, and learning parameters that control whether links between them form and how long they last. These co-evolving components shape which associations are remembered, and thus which are available to downstream nodes in the network that come to represent combinations of elements as unified entities, or ‘chunks’. Chunking enables animals to assign more context-specific meaning to combinations—for example, distinguishing ‘teacup’ from both ‘tea’ and ‘cup’—and helps build species-specific cognitive representations of the world. While Lotem and Halpern’s model is conceptual, the contribution by Leadbeater & Watrobska [9] illustrates that it captures many aspects of how neurons embody learning in the brain. They explore how memories are formed, stored and erased, emphasizing the value of a neuroscience-informed approach to understanding how learning parameters may evolve under different ecological pressures.

Lotem & Halpern [8] illustrate how chunking can pave the way for major evolutionary innovations, such as humans’ advanced ability to learn sequences. Sequence learning is a crucial feature of language, among other elements of human cognition [10–12], and is computationally challenging because the number of potential combinations that must be remembered snowballs as sequences get longer. In their contribution to this issue, Lind and Jon-And [13] question whether investment in such monumental tasks is worthwhile outside of a human context, highlighting how sequential information could be stored or sequential behavioural outputs achieved through less computationally intensive mechanisms. For example, sequences of stimuli can be approximately captured through the strength of lingering memory traces rather than precise order representation, such that for an animal exposed to a temporal sequence of stimuli ABC, the trace of C is the strongest, while B may still linger and A is the faintest. Behavioural sequences such as those used by chimpanzees to hammer nuts, or by gulls to open crisp bags, can similarly be explained without invoking precise sequence representation through well-established associative chaining, whereby one stimulus (e.g. a crisp bag) becomes a conditioned reinforcer that is rewarding in itself. In highlighting that sequence representation is potentially neither necessary nor well evidenced outside of humans, Lind and Jon-And [13] question the utility of faithful sequence representation as a framework that is useful in characterizing cognitive diversity in non-human animals for whom its cultural, language-based applications are irrelevant.

The above contributions illustrate that the lineage-specific evolutionary modifications to initial shared building blocks are integral elements of cognitive processes, and the end result is thus not well captured by a human-based cognitive glossary. But are there particular selection pressures that render certain modifications more likely to occur, and what constrains the trajectory that they can take? The contributions in §1b address these questions from the perspectives of both selection pressures that drive evolution and adaptive landscapes that make some endpoints more likely than others.

### (b) Adaptive landscapes and the shape of evolutionary trajectories

Classically, hypotheses as to what type of selection pressures underlie cognitive change are divided into ‘social intelligence hypotheses’, which propose that aspects of social life have been the primary force shaping animal cognition, and ‘ecological intelligence hypotheses’ that (despite their broad name) focus on the challenges posed by different foraging tasks [14–18]. In their contribution to this issue, Hahn *et al.* [19] highlight the statistical complexity of the social environment as both a selective force on, and an outcome of, individual decision-making processes. When animals use cognition to navigate uncertainty in social relationships, the changes in their behaviour change the social environment itself, opening up new evolutionary pathways that may differ across species, eventually generating very different societies. Hahn *et al.* [19] set out the steps that will be necessary to understand this dynamic interplay, from exploring bottom-up effects on configuration of networks following disturbance to top-down effects on network structure that influence the strength and direction of selection. Schwob *et al.* [20] are also interested in the social environment, but take an empirical approach, comparing two primate species across a battery of cognitive or behavioural assays. Despite major differences in the cooperative/despotic social styles of the focal species, these authors do not detect differences in socio-cognitive performance and conclude that in this case, divergent social contexts have not produced differences in cognitive skills. These findings highlight the need to investigate not only whether cognitive skills differ between species but also how animals use their skills to derive benefits in their natural environments. For instance, Schwob *et al.* raise the possibility that the more despotic rhesus macaques could use information about what others can see to outcompete others over access to food, while the more socially tolerant Barbary macaques might use these same skills to coordinate affiliative interactions.

Hahn *et al*.’s [19] perspective considers how evolutionary trajectories can be produced by nonlinear dynamics of the selective environment itself, but macroevolutionary feedback effects can also reflect external constraints and facilitators. For example, Teja *et al.* [21] focus on a co-evolutionary ecological intelligence hypothesis: cognitive and sensorimotor abilities allow individuals to source high-quality diets, which in turn provide the energy needed to allow for further neural expansion, which facilitates further cognitive evolution. They identify genes linked to both brain size and diet—many of which are involved in neurodevelopment and metabolism—across a set of 50 primates, and use phylogenetic path analysis to demonstrate that changes in diet quality have led to stronger selection on genes that drive changes in relative brain size in their focal group. Teja *et al.*’s implementation of phylogenetic path analysis is a means to address the challenge of establishing the probable directionality of correlational relationships, which is one of the concerns about phylogenetic correlations voiced by González-Forero and Gómez-Robles [22], alongside the possibility of spurious correlations driven by a third variable, or exaptations that evolved for reasons other than their current function. To bypass these hurdles, they advocate simulation-based inference as a powerful approach, based on a recently published mechanistic model [23–25] that tracks how energy is allocated to neural, reproductive and other somatic tissue within lifetimes, and how such lifetime energy allocation changes over generations. When applied to six hominin lines, the model captures many key patterns of brain size evolution, but illustrates that these changes are driven by developmental linkage between brain size and the true target of selection—fertility—that arises in ecologically challenging environments. In other words, their approach raises the intriguing suggestion that brain size increases in certain lineages because there is selection for fertility that has impacts for brain and body size, rather than direct selection for cognitive performance that increases survival.

González-Forero and Gómez-Robles [22] do not yet have an explanation for why fertility becomes so tightly linked to brain size, but Zwoinksa *et al.*’s contribution [26] illustrates an experimental approach to this type of question using the nematode worm *C. elegans.* This powerhouse of developmental biology and neuroscience learns to identify odours associated with the transient bacterial blooms that are its main food, and Zwoinska *et al.* [26] illustrate that manipulation of a particular conserved signalling pathway known to be associated with memory both within and across generations (i.e. epigenetic memory) has correlated consequences for both learning ability and life history. In doing so, they provide an accessible introduction to the unparalleled potential within this system and others like it (e.g. *Drosophila*) for proximate understanding of how cognition evolves in concert with life-history traits, providing the first step towards understanding why such links might exist at an ultimate evolutionary level.

In contrast to the tight linkage discussed by González-Forero & Gómez-Robles [22], Zwoinska *et al.* [26]and Hodge *et al.* [27] provide an empirical example in which evolutionary changes in one process have had little influence on another. *Heliconius* butterflies have undergone a significant expansion of their mushroom bodies that is thought to be the result of their unusual foraging ecology: feeding on pollen requires long-term memory of trapline routes between relatively distant forage plants [28]. The same research group have previously shown that *Heliconius* species have enhanced long-term visual memory, most likely because they navigate using visual cues. Here, they show that such effects are limited to visual contexts, such that *Heliconius* species perform no better than outgroup controls in olfactory associative tasks. Leadbeater and Watrobska’s contribution [9], mentioned above, explains why this might be: since visual and olfactory stimuli are represented by different neural substrates within the mushroom bodies, there is no reason why expansion in one should necessarily affect the other. Triki *et al.* [29] also focus on evolutionary changes in brain size, raising what they term the ‘fish challenge to cognitive evolution’: how is it that fish, with brains on average ten times smaller relative to body size than endothermic vertebrates, are nevertheless capable of cognitive feats similar to their warm-blooded cousins? They suggest that the primary explanation for this may be linked to an increase in sensory–motor challenges owing to the challenges of gravity, greater visibility and sound propagation following the transition from living in the water to navigating the land and the skies.

### (c) How can we detect historical or current selection?

While the correlational phylogenetic approaches mentioned above have provided one approach to the problem of identifying selection pressures, more recent years have seen the growth of an alternative approach, following Darwinian logic by examining the fitness consequences of individual cognitive variation, e.g. [30–32]. Numerous studies have now adopted this within-species approach to attempt to detect directional selection in action, typically by regressing individual performance in a particular cognitive test against fitness-relevant traits in the wild such as winter survival, clutch size or foraging success. Madden *et al.* [33] provide a quantitative review of the results of these studies, and show that the majority (70%) have failed to find any effect. There are many potential reasons for this: for instance, apparent variation in individual performance may reflect other variables (e.g. current hunger state), or there may be trade-offs between different traits and costs that counteract any directional selection. While the authors highlight the potential value of the intra-specific approach in understanding the pressures, trade-offs and constraints that shape selection across species, they also emphasize important limitations such as small sample sizes, and reporting of patterns that emerge *post hoc* as conclusive. Sol *et al.* [34] take this critical evaluation further, highlighting the need for precise characterization of functionally relevant cognitive traits, a deeper understanding of heritable variation, more robust assessments of key fitness components across large cohorts and extended time scales, and clearer identification of the fitness benefits and selective pressures involved.

Healy [35] also argues that focusing on functionally relevant cognitive traits with clear links to fitness is critically important, identifying nest-building and food-storing as contexts that potentially meet this criterion. Following an illuminating history of how research on animal cognition and its applications in behavioural ecology have developed- from the perspective of a key contributor to that story- Healy sets out the potential role of experience in the development of nest-building, providing evidence that avian parents learn what works and what does not through long-term trial and error, and modify their behaviour accordingly. In a food-storing context, Pravosudov *et al.* [36] review a programme of research on food-storing chickadees that has become the textbook example of how harsh environments select for spatial learning abilities, combining neuroanatomy, genetics, behaviour and life-history analyses in almost 30 years of work.

Pravosudov’s research programme is a uniquely deep exploration of heritable variation in an ecologically relevant cognitive trait that is strongly linked to fitness outcomes, but implementing such approaches in other systems is fraught with logistical challenges. Sheehan and Miller [37] set out a powerful and long-awaited complementary means to detect the action of selection on cognitive traits by searching for evidence of selection on cognitive genes in the genomes of extant species—a population genetics approach to cognitive evolution that is long overdue given the availability of high-quality, high-coverage sequencing that has now been within reach for quite some time. These authors explain how such data can answer questions about the existence, rate and trajectory of cognitive evolution, exemplified by their work on a lineage of Polistine wasps where the evolutionary emergence of facial recognition has been accompanied by rapid recent selection for visual processing abilities. This is a genuinely integrative contribution that, through both accessible review of the relevant population genetic concepts and careful explanation of their application to cognitive evolution, promises to bring a powerful tool to the field.

## 2. Conclusions

We feel that comparative cognition is shifting its perspective. Looking for a hierarchy of ‘basic’ to ‘complex’ human-like processes that might be detectable in other species if they could only be isolated from sensory systems is no longer the key focus; as Barton & Barrett [7] point out, that approach produced decades of unresolved arguments. Instead, the field is now poised to deliver a more biologically grounded view. This emerging perspective re-embeds cognitive processes within the context of the whole animal. It treats each lineage as its own evolutionary endpoint. It also rejects the artificial dichotomy between ‘social’ and ‘ecological’ intelligence in favour of a more nuanced understanding that cognitive challenges in the wild—whether social or ecological—are multifaceted and interconnected. Central to this progress are a growing diversity of approaches described in the articles that follow, which span theoretical modelling [8], neurogenetics [9], neuroanatomy [29,38], population genetics [39], experimental evolution [40–43], field experiments [19,35,36], and more. The value of those new approaches, and of their intersection with the fields from which they derive, is sometimes in the ideas that they generate. How could we have known, for example, that insects might have the option to direct unreliable memories into energetically cheaper processes until neuroscience told us that those alternative options exist [40,41]? Sometimes it is in the experimental possibilities that open up: how exciting to be able to control selection and observe the results, as is possible for experimental evolution models [42–44], or to manipulate cognitive genes and observe pleiotropic consequences for life-history traits [26]. And sometimes it is in the scale of possibilities: whole genomes contain histories of selection that could otherwise never be accessed [39]. We think these are exciting times for understanding the evolution of animal minds.

## Data Availability

This article has no additional data.

## Footnotes

^1^
We embrace Shettleworth’s classic broad-sense definition of cognition as all the ways in which animals take in information through the senses, process it, retain it and decide to act on it [3], because this definition is not derived from an anthropocentric viewpoint and thus encompasses the full spectrum of cognitive diversity that may exist in the animal kingdom.
